# Effects of spatial accessibility of community health services on the activities of daily living among older adults in China: a propensity score matching study

**DOI:** 10.3389/fpubh.2024.1335712

**Published:** 2024-06-12

**Authors:** Yuee Li, Xiaohui Liu

**Affiliations:** School of Humanities and Law, Northeastern University, Shenyang, China

**Keywords:** community health services, spatial accessibility, activities of daily living, propensity score matching, older adults

## Abstract

**Background:**

The Chinese government proposes to establish a hierarchical diagnosis and treatment system, and attaches great importance to community health services. Under the background of population aging and the increase of older adults with disability, this study aimed to analyze the effect of spatial accessibility of community health services on the activities of daily living (ADL) among older adults in China.

**Methods:**

A research sample of 7,922 older adults from the Chinese Longitudinal Healthy Longevity Survey (CLHLS) data in 2018 was adopted. There were 2,806 participants in the treatment group and 5,116 participants in the control group. The propensity score matching method was adopted to match the treatment and control groups to calculate the values of average treatment effects on treated (ATT).

**Results:**

The results of kernel density matching method showed that the factual ADL score of the treatment group was 10.912, the counterfactual ADL score of the control group was 10.694, and the ATT value was 0.218 (*p* < 0.01). The spatial accessibility of community health services could significantly improve the activities of daily living among older adults in China. Meanwhile, there was urban-rural heterogeneity in the impact of spatial accessibility of community health services on the activities of daily living of older adults in China. The effect value in urban samples (ATT = 0.371, *p* < 0.01) was higher than that in rural samples (ATT = 0.180, *p* < 0.01).

**Conclusion:**

Spatial accessibility of community health services could improve the activities of daily living among older adults in China. The Chinese government should take actions to improve the distribution of community health service resources.

## 1 Introduction

According to data from the seventh National Census, the population aged 60 and above in China counted for more than 264 million people in 2020, accounting for 18.70% of the total population, and the population aged 65 and above counted 190 million people, accounting for 13.5% of the total population (1). China will soon be a super aging society, according to the international standard in which those aged 65 and above account for over 14% of the total population (2). According to the results of the fourth sample survey on the living conditions of senior citizens aged 60 and above in urban and rural China jointly released by the China National Committee On Aging, Ministry of Civil Affairs and Ministry of Finance, the number of disabled and semi-disabled older adults in 2015 was approximately 40.63 million, accounting for 18.3% of the total older population in that year (3). One study predicted that the disabled older population in China would reach 77.66 million in 2030 (4). The proportion of disabled older people in the total older population in China will continue to rise.

Due to the aging of the population, the rapid growth of residents’ basic health needs, and the unreasonable allocation of medical and health service resources, Chinese residents are inconvenient to seek medical treatment, and the burden of medical expenses is increasing. In order to solve this problem, the Chinese government issued policies in 2015 aimed at establishing a hierarchical diagnosis and treatment system. Hierarchical diagnosis and treatment refers to the classification according to the severity of the disease and the difficulty of treatment, and different levels of medical institutions undertake the treatment of different diseases. It is simply summarized as “first diagnosis at the grassroots level, two-way referral, acute and chronic treatment, linkage between upper and lower levels” (5).

In the context of China’s hierarchical diagnosis and treatment system reform, community health services, as one of the important components of primary health services, has been paid more and more attention. On the one hand, the number of community health service providers has increased. With the reform of China’s health service system, private community health service centers play a complementary and increasingly important role in providing basic health services to residents (6). The increase in the number of service providers still cannot solve the problem of unbalanced utilization of community health services between urban and rural areas (7, 8). On the other hand, residents’ medical behavior has also changed. More than 62.2% of community residents choose to receive initial diagnosis and treatment in the community (9). But the utilization rate of community health services varies among different groups. Some studies found that disability and illness were the main determinants of using community health services (10, 11). The World Report on Disability and People with Intellectual Disabilities highlighted the need for people with disabilities to have access to adequate health services in the community (12). However, there were a significant number of people with disabilities in China who still did not have access to health services in communities to meet their needs for activities of daily living (13).

Therefore, under the background of China’s hierarchical medical system reform and the increasing number of older adults with disabilities, it is of great importance to explore the effect of spatial accessibility of community health services on the activities of daily living among older adults in China. This study adopts the propensity score matching method to carry out a quasi-experimental study on the effect of spatial accessibility of community health services on the activities of daily living among older adults (age 60 and above) and calculate its effect value. The results of this study help to reveal the beneficial effects of attaching importance to the construction of community health service institutions in the context of the establishment of the hierarchical diagnosis and treatment system in China.

## 2 Literature review

### 2.1 Activities of daily living among older adults

Activities of daily living (ADL) are important indicators for evaluating the health status of the older people. The literature on ADL ability of older people focuses on two aspects. The first is the study of the measurement scale of ADL ability. In previous studies, scholars have developed a variety of measurement tools for ADL ability. Katz Index (14), Barthel index (15), Pfeffer’s Functional Activity Questionnaire (16), and Lawton and Brody’s Activities of Daily Living scale (17) were widely used. The second is the study of the influencing factors of ADL ability. For example, factors such as age, education level, depression, chronic diseases, living alone, air quality, dietary habits, drinking habits, social activities, sports activities, and housing accessibility were all important factors affecting the ability of older people to perform activities of daily living (18–25). Some researchers put forward a series of measures to improve the ADL ability of older adults. Chen et al. suggested that measures such as training informal caregivers, improving team-based primary care, and using intelligent software could improve ADL ability (26). And Burge et al. believed that regular physical exercise was an effective measure to improve ADL ability (27).

### 2.2 The accessibility theory

The accessibility theory comes from transportation geography, with the aim of analyzing the convenience of the public in accessing public resources. Accessibility is divided into spatial accessibility and non-spatial accessibility (28). Space accessibility is mainly influenced by distance factors; non-spatial accessibility is affected by factors such as the public’s economic income, service preference and and size of service organizations (29). Nowadays, the accessibility theory has been widely applied in traffic geography to evaluate and improve the accessibility of transportation in certain cities or areas (30–32).

And this theory was introduced into the field of public health to assess the inequality in public access to healthcare services. Based on the spatial accessibility measurement method, Kalogirou believed that compared to older or low educated residents, younger, working age population as well as people with the highest educational attainment have better accessibility to public hospitals in Greece (33). Bauer et al. found a high correlation between the accessibility of German hospitals and the level of urbanization, and the accessibility of general inpatient care was either high or at least not significantly low (34). Martinez and Rojas analyzed that in Concepcion metropolitan area of Chile, about 4.1% of the population had unfavourable or very unfavourable accessibility to public hospitals (35). In conclusion, based on the accessibility theory, existing studies have explored the current status of access to health care services and its associated factors in various regions or cities.

### 2.3 The spatial accessibility of community health services

Community health services are an important part of community services (36). It provides prevention, medical treatment, health care, rehabilitation and health education services for residents (37). The spatial accessibility of community health services refers to the convenience in distance when the public obtains the above community health services. Studies on spatial accessibility of community health services focus on two aspects. On the one hand, the existing studies have analyzed the spatial accessibility level of community health services. Evans et al. based on data from the Health Resources and Services Administration in the United States, found that community health center accessibility substantially increased from 2008 to 2016 (38). By using a geographic information system database, Vannier et al. concluded that in Christchurch – Otautahi, New Zealand, the farther away from the city center, the less community health services were available, but overall, there was a high level of spatial accessibility (39). Oliphant et al. also analyzed local geographical data and believed that the level of spatial accessibility of community health services in Niger increased significantly from 2000 to 2013, but the current level of spatial accessibility was still not high (40). On the other hand, the existing research has proposed some improvement measures to improve the level of accessibility. These measures include: reasonably layout community health service institutions to avoid excessive concentration; formulate corresponding subsidy policies to attract high-quality medical and health resources; Increase multiple modes of transportation to promote convenient travel (41, 42).

In summary, the existing literature pays little attention to the relationship between spatial accessibility of community health services and human ADL capacity. Although some scholars have analyzed the impact of community health services on the physical and mental health of older adults (43, 44), the methods used were mostly traditional regression models. The influence effect value obtained by traditional regression model analysis could be affected by other confounding variables. In addition, based on the accessibility theory, it is of practical significance to pay attention to the spatial accessibility of community health services. In view of the increasing number of older adults with disability in China, using non-traditional regression methods, analyzing the relationship between spatial accessibility of community health services and the daily living ability of older adults was the most basic purpose of this study.

## 3 Materials and methods

### 3.1 Study design

This is a cross-sectional study to examine the impact of spatial accessibility of community health services on the activities of daily living of older people in China. The activities of daily living of older people may be affected not only by the spatial accessibility of community health services, but also by factors such as dietary habits, exercise, and sleep quality. The latter variables are called confounding variables (45). The effect value of the spatial accessibility of community health services on the activities of daily living among older adults obtained by traditional regression analysis is not pure due to the influence of confounding variables. Therefore, the propensity score matching method was used to eliminate the influence of confounding variables on the activities of daily living of older people, comparing the pure effect values of the effect of spatial accessibility of community health services on ADL scores among older adults. Community health services were treated as a natural intervention, avoiding the biased estimates resulting from the problem of endogeneity caused by confounding variables.

### 3.2 Data source and sample selection

This study used data from the Chinese Longitudinal Healthy Longevity Survey (CLHLS). This is a publicly available dataset compiled by Peking University’s Center for Healthy Aging and Development Studies (CHADS). Participants were randomly selected from half of the counties and cities in 23 provinces, covering about 85 percent of China’s total population. The CLHLS baseline survey aimed to investigate the determinants of human longevity. Therefore, in the sampled counties and cities, CLHLS attempted to interview all centenarians who volunteered to participate. The CLHLS adopted a targeted random sample design to ensure representativeness, establishing sampling weights based on age, sex, and place of residence (i.e., near the residence of centenarians) (46). Due to this sampling design, the average age of older participants was older (43).

Currently, there are eight waves of survey data in CLHLS. The CLHLS baseline survey began in 1998, and participants were followed up in 2000, 2002, 2005, 2008, 2011, 2014, and 2018. During the follow-up period, many participants were lost to follow-up, mainly due to address changes (47). Thus, in each wave of the CLHLS survey, new participants were interviewed. Extensive data was collected by CLHLS, including healthcare, social activities, diet, smoking, drinking, psychological characteristics, economic resources, family structure, self rated health, self rated life satisfaction, etc. (48). The CLHLS dataset had been reviewed by the Ethics Committees of Peking University. The survey respondents gave informed consent before participation.

This is a cross-sectional study, and the latest data in the CLHLS database was still survey data from 2018. Thus, we selected the 2018 CLHLS data as the sample data source for this study. A research sample of 7,922 older adults from the CLHLS data in 2018 was adopted.

### 3.3 Variable selection and measurement

#### 3.3.1 Activities of daily living among older adults

The dependent variable of this study was the activities of daily living (ADL) among older adults, which was assessed by the Katz scale. The Katz scale includes six indicators (bathing, dressing, toileting, transferring, continence, and feeding), each with three categories that evaluate participants: “0 = completely dependent,” “1 = partially dependent” and “2 = completely independent” (14). The total score ranges from 0 to 12 points. The higher the score, the higher the ADL ability of older adults.

Before data analysis, the reliability and validity of Katz scale in the samples were tested. Firstly, the reliability of Katz scale was analyzed. By analysis, in the sample, the Cronbach’s α coefficient of the Katz scale was 0.866, indicating good internal consistency. Secondly, the validity of Katz scale was analyzed. Exploratory factor results showed that the Kaiser-Meyer-Olkin (KMO) value of the Katz scale was 0.860 and the Barlett sphericity test was significant (*p* < 0.05). Based on principal components methods, the factor had an eigenvalue of 3.62, accounting for an accumulated total of 67.3% of the variance. All of the ADL items loaded on the factor with a correlation of 0.5 or greater. The solution extracted only one factor so the solution was not rotated. In addition, the confirmatory factor analysis of the Katz scale was carried out by using the maximum likelihood robust estimation method. The results showed that the root mean square error approximation (RMSEA) value was less than 0.1, the 90% confidence interval value was less than 0.08, the comparison fitting index (CFI) and tucker-lewis index (TLI) were greater than 0.9, and the standardized residual mean root (SRMR) value was also less than 0.08 (RMSEA = 0.065, 90% CI = 0.053–0.077, CFI = 0.985, TLI = 0.975, SRMR = 0.025). The analysis results showed that the Katz scale had good construct validity.

#### 3.3.2 Spatial accessibility of community health services

The treatment variable in this study was the spatial accessibility of community health services for older adults. The propensity score matching method requires that the treatment variable must be divided into two categories. We assigned treatment and control groups according to whether health services were available in the community where older adults lived. The treatment group referred to older adults in their community with community health services. The control group was older adults without community health services in their community. Therefore, the variable was treated as 0 = inaccessibility and 1 = accessibility.

#### 3.3.3 Control variable

Demographics, health behavior, and other community services characteristics were selected as control variables (49). Demographic characteristics were measured with five indicators, including sex (0 = female, 1 = male), age (continuous variable, 60–117 years old), area (0 = rural, 1 = urban), spouse (0 = not alive, 1 = alive) and income level (0 = poor, 1 = rich). Health behaviors were measured by eight variables, including regular consumption of vegetables (0 = not eating frequently, 1 = eating frequently) and fruits (0 = not eating frequently, 1 = eating frequently), smoking (0 = non-smoking, 1 = smoking), exercise (0 = not exercising regularly, 1 = exercising regularly), leisure activities (0 = infrequent, 1 = frequent), sleep quality (0 = not good, 1 = good) and an annual physical examination (0 = none, 1 = yes). Other community services variable was measured by only one indicator, which included two options: 0 = none and 1 = yes.

### 3.4 Statistical analysis

STATA 16.0 was used for data analysis. Firstly, descriptive statistics were used to show the basic characteristics of samples. Secondly, the multiple linear regression method was used to analyze the factors affecting the outcome variables, and the non-significant related variables were eliminated. Thirdly, we used the probit model to calculate the propensity score of each variable, tested the matching effect of each variable before and after matching, calculated the average treatment effects on treated (ATT) obtained by using the kernel density matching method, and analyzed whether there was urban-rural heterogeneity in the influence effect. Finally, the radius matching method was used to test the robustness of the results.

## 4 Results

### 4.1 Descriptive statistical results

Table 1 shows the basic characteristics of the participants. The sample contained information on 7,922 participants, including 2,806 participants in the treatment group and 5,116 participants in the control group. The mean value of the variable of spatial accessibility of community health services was 0.35 ± 0.48. The average ADL score for all older participants was 10.81 ± 2.51, which was close to the maximum of 12. In addition, the mean ADL score of the treatment group (10.87 ± 2.43) was higher than that of the control group (10.70 ± 2.63). The descriptive statistics of the other variables are shown in Table 1.

**Table 1:** Descriptive statistical results of variables.

Variable	Mean ± SD		
	Total	Treatment group	Control group
ADL score	10.81 ± 2.51	10.87 ± 2.43	10.70 ± 2.63
Spatial accessibility of community health services	0.35 ± 0.48	/	/
Sex	0.56 ± 0.50	0.55 ± 0.50	0.57 ± 0.50
Age	85.32 ± 11.70	85.44 ± 11.74	85.25 ± 11.69
Area	0.32 ± 0.47	0.27 ± 0.45	0.34 ± 0.47
Spouse	0.42 ± 0.49	0.42 ± 0.49	0.41 ± 0.49
Income level	0.20 ± 0.40	0.20 ± 0.40	0.19 ± 0.40
Vegetables	0.11 ± 0.32	0.23 ± 0.42	0.05 ± 0.21
Fruits	0.47 ± 0.50	0.46 ± 0.50	0.47 ± 0.50
Smoking	0.16 ± 0.36	0.29 ± 0.46	0.08 ± 0.28
Exercise	0.32 ± 0.47	0.28 ± 0.45	0.34 ± 0.47
Sleep quality	0.53 ± 0.50	0.53 ± 0.50	0.52 ± 0.50
Leisure activities	0.75 ± 0.43	0.76 ± 0.43	0.75 ± 0.43
Physical examination	0.66 ± 0.47	0.74 ± 0.44	0.62 ± 0.49
Other community services	0.41 ± 0.49	0.63 ± 0.48	0.30 ± 0.46
N	7,922	2,806	5,116

### 4.2 Multivariate analysis results

Before propensity score matching, a multiple linear regression was used model to analyze the significant correlation between the multiple variables and the outcome variables. Table 2 shows the results of the multiple linear regression model. The pseudo-R-square value of the model was 0.2525. The spatial accessibility of community health services as the treatment variable was a positive factor affecting the activities of daily living of older people (*p* < 0.01). Some control variables, such as age, area, income level, vegetables, fruits, smoking, sleep quality, leisure activities, physical examinations, and other community services, also showed a significant correlation with outcome variables (*p* < 0.05). The above control variables were considered confounding variables in this study. In order to calculate the pure effect of spatial accessibility of community health services on ADL ability of older adults, the influence of these variables should be eliminated by propensity value matching method. In addition, in the multiple linear regression model, sex and spouse variables did not significantly affect the outcome variables (*p* > 0.05). These two variables would not be used as matching variables.

**Table 2:** Multiple linear regression model results.

Variables	B	S.E.	t	*p* > *t*	[95% CI]	
Spatial accessibility of community health services	0.188	0.055	3.44	0.001	0.081	0.295
Sex	0.011	0.056	0.19	0.848	−0.099	0.120
Age	−0.056	0.003	−20.20	0.000	−0.061	−0.050
Area	0.634	0.056	11.24	0.000	0.523	0.744
Spouse	0.014	0.062	0.22	0.828	−0.109	0.136
Income level	0.152	0.063	2.40	0.016	0.028	0.276
Vegetables	0.579	0.081	7.18	0.000	0.421	0.737
Fruits	0.274	0.053	5.20	0.000	0.171	0.377
Smoking	−0.159	0.075	−2.12	0.034	−0.307	−0.012
Exercise	0.678	0.056	12.01	0.000	0.568	0.789
Sleep quality	0.153	0.050	3.07	0.002	0.055	0.250
Leisure activities	1.154	0.065	17.86	0.000	1.027	1.281
Physical examination	0.470	0.055	8.55	0.000	0.362	0.578
Other community services	0.149	0.052	2.88	0.004	0.048	0.251
_cons	−5.174	0.260	−19.90	0.000	−5.684	−4.665

### 4.3 Propensity score matching analysis results

#### 4.3.1 Propensity score results

The probit model was used to calculate the matching propensity scores (50). Table 3 shows the variables and estimation results included in the probit model. The log likelihood value of the model was −4375.0355, and the pseudo R squared value was 0.1504. The probability values of all variables in the probit model were used as propensity scores.

**Table 3:** Estimated results of propensity score model.

Variables	B	S.E.	z	*p* > z	[95% CI]	
Age	0.003	0.002	2.22	0.027	0.000	0.006
Area	−0.361	0.037	−9.73	0.000	−0.433	−0.288
Income level	0.047	0.040	1.18	0.238	−0.031	0.126
Vegetables	0.769	0.056	13.75	0.000	0.660	0.879
Fruits	−0.051	0.033	−1.57	0.117	−0.115	0.013
Smoking	0.526	0.048	10.92	0.000	0.431	0.620
Exercise	−0.183	0.036	−5.05	0.000	−0.254	−0.112
Sleep quality	0.030	0.031	0.95	0.341	−0.032	0.091
Leisure activities	−0.014	0.040	−0.36	0.722	−0.093	0.064
Physical examination	0.377	0.035	10.62	0.000	0.307	0.446
Other community services	0.675	0.032	20.99	0.000	0.612	0.738
_cons	−1.222	0.153	−7.97	0.000	−1.522	−0.921

#### 4.3.2 Matching effect test results

The kernel density matching method was used to calculate the ATT value. The psmatch2 command in STATA 16.0 was used for the kernel density matching method (51). Before calculating the ATT value, a matching effect test was required to determine whether the matching result could be regarded as a counterfactual result (52).

The matching effect test consists of two steps. First, the treatment and control groups had to have a common support domain (53). If not, this indicated that the two groups were not comparable at all, and propensity value matching analysis could not be performed. In the kernel density matching result, 2,799 samples in the treatment group (*n* = 2,806) and 5,110 samples in the treatment group (*n* = 5,116) met the common support domain requirements. The results showed that the treatment and control groups in the sample had a large common support domain. Second, balance test of matching variables was required. The results of the matching balance test between the treatment and control groups need to be examined. A smaller absolute value of the standard bias indicates a better match. It is generally believed that as long as the absolute value of the standard bias is less than 20%, it will not lead to matching failure (54). In addition, the *t*-test was performed on the matched variables in the treatment and control groups to determine whether there were significant differences between the two groups in these matched variables. If not, the matching effect could be considered to meet the requirements.

Table 4 shows the balance test results for kernel density matching variables. Table 5 shows the overall comparison results between unmatched and matched. The results of the balance test showed that the absolute value of the standard bias of all variables in kernel matching did not exceed 20%, and the mean bias was also small. The *t* test showed that there were no significant differences between groups for all variables after matching (*p* > 0.05). The pseudo R squared values was close to zero, and the probability values of the LR chi2 test were not significant (*p* > 0.05). This finding indicated that after propensity value matching, the control variable no longer had a significant effect on the activities of daily living among older adults. Therefore, the balance test was passed.

**Table 4:** Balance test results for kernel density matching variables.

Variable	Unmatched	Mean		% Bias	% Reduct	*t*-test	
	Matched	Treated	Control		Bias	*t*	*p* > t
Age	U	85.439	85.251	1.6		0.68	0.495
	M	85.415	85.593	−1.5	5.3	−0.57	0.568
Area	U	0.272	0.341	−15.0		−6.31	0.000
	M	0.273	0.270	0.7	95.3	0.27	0.784
Income level	U	0.204	0.194	2.4		1.04	0.299
	M	0.203	0.203	0.2	93.4	0.06	0.952
Vegetables	U	0.231	0.047	55.4		25.98	0.000
	M	0.229	0.222	2.1	96.3	0.62	0.537
Fruits	U	0.463	0.469	−1.1		−0.46	0.643
	M	0.464	0.455	1.9	−73.9	0.71	0.478
Smoking	U	0.292	0.084	55.0		25.16	0.000
	M	0.290	0.279	2.9	94.8	0.90	0.370
Exercise	U	0.285	0.335	−10.9		−4.62	0.000
	M	0.285	0.269	3.6	67.0	1.39	0.164
Sleep quality	U	0.532	0.522	2.1		0.91	0.360
	M	0.531	0.534	−0.6	70.1	−0.24	0.810
Leisure activities	U	0.757	0.753	1.0		0.42	0.677
	M	0.758	0.749	2.2	−124.5	0.82	0.412
Physical examination	U	0.739	0.617	26.3		11.05	0.000
	M	0.739	0.734	0.9	96.4	0.37	0.711
Other community services	U	0.630	0.296	71.0		30.47	0.000
	M	0.629	0.625	0.8	98.9	0.28	0.779

**Table 5:** Overall comparison results between unmatched and matched.

Sample	Ps *R*^2^	LR chi2	*p* > chi2	MeanBias	MedBias
Unmatched	0.150	1548.69	0.000	22.0	10.9
Matched	0.000	3.28	0.986	1.6	1.5

Ps *R*^2^ represents Pseudo R square, LR chi2 represents chi-square test statistics, *p* > chi2 represents the probability greater than the critical value of chi-square distribution, MeanBias represents the mean of bias, and MedBias represents the median of the bias.

#### 4.3.3 Kernel density matching analysis results

Table 6 shows the results of kernel density matching for the ADL scores of older adults. Before matching, the ADL score of the treatment group was 10.866, the ADL score of the control group was 10.695, and the ATT value was 0.171 (*p* < 0.01). After matching, the factual ADL score of the treatment group was 10.912, the counterfactual ADL score of the control group was 10.694, and the ATT value was 0.218 (*p* < 0.01). The results of kernel density matching method showed that the spatial accessibility of community health services could significantly improve the activities of daily living among older adults in China. And due to the elimination of the influence of confounding variables, the ATT value after matching was higher than the ATT value before matching. It indicated that the real influence value of spatial accessibility of community health services on ADL ability of older people was much higher than the influence coefficient value in multivariate regression model. Therefore, using the traditional regression method, we could not get the real effect value of the influence. In summary, the results of kernel density matching method showed that the spatial accessibility of community health services could significantly improve the activities of daily living among older adults in China.

**Table 6:** Kernel density matching results of ADL scores among older adults.

Sample		Treated	Controls	ATT	S.E.	T
Total	Unmatched	10.866	10.695	0.171**	0.059	2.91
	Matched	10.912	10.694	0.218**	0.066	3.30

* *p* < 0.05, ** *p* < 0.01.

#### 4.3.4 Urban-rural heterogeneity analysis results

On the basis of the effect of the treatment variables on the outcome variables, we further analyzed whether there was urban-rural heterogeneity in the influence relationship. Table 7 shows the results of urban-rural heterogeneity analysis after kernel density matching. In the urban sample, the ADL score of the treatment group was 10.741, the ADL score of the control group was 10.370, and the ATT value was 0.371 (*p* < 0.01). In the rural sample, the ADL score of the treatment group was 10.995, the ADL score of the control group was 10.815, and the ATT value was 0.180 (*p* < 0.01). In both groups of samples, the spatial accessibility of community health services had a significant positive impact on the ADL ability of older adults. However, the ATT value in urban samples was higher than that in rural samples. Therefore, there was urban-rural heterogeneity in the impact of spatial accessibility of community health services on the activities of daily living of older adults in China.

**Table 7:** Urban-rural heterogeneity analysis results after kernel density matching.

Sample		Treated	Controls	ATT	S.E.	T
Matched	Urban	10.741	10.370	0.371**	0.133	2.80
	Rural	10.995	10.815	0.180**	0.075	2.39

* *p* < 0.05, ** *p* < 0.01.

### 4.4 Robustness test results

By changing the matching method, we used the radius matching method to test the robustness of the results. Table 8 shows the robustness test results of this study. In the total sample, the ATT value calculated by the radius matching method was 0.224 (*p* < 0.01). The result was very close to the ATT value calculated by the kernel density matching method. In addition, the ATT values in urban samples and rural samples were also similar to the original results. And the results of radius matching also showed that the spatial accessibility of community health services had urban-rural heterogeneity on the ADL ability of older adults. In conclusion, the results obtained in this study could be regarded as robust.

**Table 8:** Robustness test results.

Sample		Treated	Controls	ATT	S.E.	T
Matched	Total	10.918	10.694	0.224**	0.066	3.38
	Urban	10.734	10.370	0.364**	0.133	2.75
	Rural	11.002	10.815	0.187**	0.075	2.48

* *p* < 0.05, ** *p* < 0.01.

## 5 Discussion

CLHLS 2018 survey data were adopted to analyze the effect of spatial accessibility of community health services on the activities of daily living among older adults in China. The results of the mean ADL score of older adults obtained by descriptive statistics in this study show that the older adults in China are mostly non-disabled or mildly disabled, and the proportion of older adults with moderate and severe disability is relatively low. This is consistent with the findings of a published study (21). In addition, through descriptive statistical analysis, we found that the majority of older people in China lived in communities without health services. Similar to China, Oliphant et al. point out that community health services were not available to many people in Niger (40). However, in Christchurch-Ottahi, New Zealand, local residents had easy access to community health services (39).

Prior to propensity value matching, a multiple linear regression model was used to analyze whether each control variable was significantly associated with ADL ability of older adults. The results showed that higher income levels, frequent consumption of vegetables, good sleep quality, regular exercise, frequent leisure activities, annual physical examinations, and community services in the community were positive factors affecting the ADL ability among older adults, while smoking was negative factors. These results are consistent with the results of several studies that have analyzed factors affecting ADL ability in older adults (18–24). However, one study found that housing accessibility is also an influential factor in activities of daily living (25), which is a variable not considered in our study.

We used the kernel density matching method to match the treatment and control groups to calculate the pure effect value. The results of the kernel matching method showed that the ADL score of older adults affected by the space accessibility of community health services was higher than that of older adults affected by the space inaccessibility of community health services. The spatial accessibility of community health services could significantly improve the activities of daily living among older adults in china. Some scholars have analyzed the impact of community health services on human health. The identified beneficial effects could be divided into two aspects: On the one hand, it helps to improve physical health, such as reducing the time patients spend in bed (55), alleviating joint pain (56); On the other hand, it may improve the mental health of the public, especially the older group (43, 44). Consistent with the views of these scholars, this study also believes that community health services can effectively improve the health status of older adults. However, this paper focused on the convenience of public access to community health services in spatial distance, and analyzed whether this convenience would have a significant impact on the ADL ability of older adults.

Through further analysis, the study found that the spatial accessibility of community health services has urban-rural heterogeneity on the ADL ability of older adults in China. There are differences in the supply and distribution of community health service resources between urban and rural areas of China, and most of them are distributed in urban areas (57, 58). Therefore, compared with older adults in rural areas, older adults living in urban areas are more likely to access community health services. In addition, the community health service level in urban areas is higher, with more comprehensive service items and facilities (59). The health of older people in urban areas is more likely to be safeguarded by community health services. The data analysis results show that compared to rural areas, the effect value of spatial accessibility of community health services on the ADL ability of older people is higher in urban areas. This conclusion demonstrates the negative impact caused by the uneven supply and distribution of community health service resources between urban and rural areas.

## 6 Conclusion

Compared with the traditional regression analysis method, this study analyzed the pure effect value of the spatial accessibility of community health services on the ADL ability among older adults in China based on propensity score matching method. The spatial accessibility of community health services could improve ADL ability among older adults in china. Due to the uneven distribution of community health service resources between urban and rural areas, there are urban-rural differences in the effect of the spatial accessibility of community health services on the activities of daily living among older adults.

Several actions should be considered to improve the spatial accessibility of community health services.

Firstly, the resources of community health service institutions should be reasonably distributed to avoid excessive concentration. In areas with fewer community health service institutions, especially in rural areas, emphasis should be placed on the construction of community health service institutions. In areas with dense distribution of community health service institutions, excess community health service resources should be transferred to areas with scarce resources.

Secondly, targeted financial support policies should be formulated to increase the number of community health service institutions in rural areas. The Chinese government has proposed the social development goal of “equalization of basic public health services for urban and rural residents,” with the aim of eliminating the huge differences in basic public health service resources between urban and rural areas in China (60). Therefore, local governments need to intervene to increase the number of community health service institutions in rural areas. Therefore, intervention should be implemented through the formulation of targeted financial support policies to increase the number of community health service institutions in rural areas.

Thirdly, multiple modes of transportation should be developed to improve the convenience of residents in accessing community health services. If there are diverse modes of transportation around the community, residents can quickly reach the community health service institutions they want to go to. Therefore, we propose to develop various modes of transportation near the community, such as bus, subway, light rail, airplane, high-speed rail and so on.

Fourthly, services such as home hospital beds and home visits should be emphasized. Some special groups of older people, such as older people with disabilities, are not convenient to reach service institutions because of the difficulty in activities of daily living. Therefore, the development of these services will help to meet the health care needs of older people, especially those with disabilities.

In addition to the above measures, for older people and their families, they can migrate to cities or communities with high accessibility to community health services. It is undeniable that compared to economically underdeveloped cities, economically developed cities have more community health service resources. And health service resources also vary between communities. By moving to these cities or communities with more health service resources to meet the health care needs of older persons, especially those with disabilities.

This study has some limitations. Firstly, CLHLS only surveyed 23 provinces in China. Other provinces, such as Tibet, Qinghai, Xinjiang and Gansu, were not included in the survey. Therefore, it is unclear whether the results of this study are applicable to these areas that have not been surveyed. Secondly, since the latest data from the database survey has not been released for a long time, only the data from 2018 can be used. In recent years, the development of community health services in China may have changed. How these changes affect the ADL ability of older adults cannot be further studied in this paper. Thirdly, although some relevant variables were selected as confounding variables in this study, not all potential confounding variables were included, for example, factors such as chronic diseases and home care services. Fourthly, the propensity score matching method has stricter premise requirements, and these assumptions may not be fully fulfilled in daily life. The research results of this paper can only prove the effect of community health services on the activities of daily living among older adults in China in a theoretical sense. Therefore, future studies should be conducted in these provinces that have not been surveyed by the CLHLS and analyze the effects of other potentially confounding variables on ADL ability. In addition, a new wave of CLHLS surveys should be conducted to obtain the latest data. Finally, future research needs to focus on how to improve the distribution of community health services resources.

## Data availability statement

The original contributions presented in the study are included in the article/supplementary material, further inquiries can be directed to the corresponding author.

## Ethics statement

The CLHLS dataset are publicly available and open to researchers all over the world. The current study is a secondary analysis of the deidentified CLHLS dataset. This dataset neither involves the physical integrity of subjects nor deviates from the principle of informed consent. The authors utilized the CLHLS dataset that has been reviewed by the Ethics Committees at the data collection and compilation institution. The authors did not merge or enhance the dataset in any way that could lead to the identification of study participants. Therefore, further review by the Ethics Committees was not required.

## Author contributions

YL: Writing – review & editing, Writing – original draft. XL: Writing – review & editing, Writing – original draft.
